# The Treatment of Fractures from a Common-Sense Point of View

**Published:** 1907-10-12

**Authors:** 


### Points in Surgery.

### THE TREATMENT OF FRACTURES FROM A COMMON-SENSE POINT OF VIEW.

### VIII.—FRACTURE OF THE OLECRANON.

Fracture of the olecranon process is not a very common injury: it is not met with so frequently as might be expected, when one considers that the olecranon is a structure superficially placed and so much exposed to the different forms of direct violence, and that it is attached to the shaft of the ulna by a constricted base which would, it might be thought, form a weak spot. The truth is that its sharp conformation protects it from more frequent damage, because the force producing the violence tends to slide off it. All bony points which are exposed to sudden violence are similarly sharp—the front of the tibia is another very good example—while those which are subjected to constant pressure, like the calcaneum, are rounded. Even this natural protection, however, is insufficient to prevent the olecranon from being fractured occasionally. The injury may be produced by direct or by indirect violence, or even by sudden over-action of the triceps muscle when the arm is bent at a right angle, just as the patella may be broken by over-action of the quadriceps extensor. The immediate result is inability to actively extend the arm. If the direction of the force producing the fracture is continued there may be dislocation forwards of the upper ends of radius and ulna, a complication which is not in itself of much moment, except that when this malposition has once been established it tends to recur during treatment. There is no doubt that in the absence of any complete contraindication to operation, such as serious organic disease, the correct line of treatment in these cases is to perform an open operation, and to wire the fragments. Such an operation in these days of aseptic surgery entails little or no risk to the surgeon, and is a better alternative than the almost certain loss of power which results from conservative treatment. Operation is especially strongly indicated if there is much separation of the fragments, and in this respect these cases vary largely; if the fibrous tissue round the bone remains intact there may be little or no displacement, but if it, too, is torn there may be a considerable interval between the fragments, the upper of which is dragged up by the triceps. When this is the case, and conservative treatment by splints and retentive apparatus is adopted, the interval is filled with fibrous tissue, and much loss of power results. The operation is moderately easily performed. The patient should lie on his back with the arm abducted from the side and flexed to its full extent. The surgeon should stand between the arm and the body of the patient in dealing with the left arm, but between the arm and the head when dealing with the right. A longitudinal incision, four or five inches long, is made over the olecranon. In all operations where bones are to be wired a free incision should be made so that the field of operation may be well defined. Some authorities recommend a horseshoe shaped flap convex upwards, but this seems to have no special advantages over the other, which gives a quite sufficiently good exposure. When the fragments are exposed, all blood clots are washed from between them with an irrigator, and the joint is washed out. The bones are next drilled with a bradawl. Each fragment is drilled from its external to its fractured surface, and the direction of the drill holes should be so managed that the two orifices on the fractured surfaces should be exactly together when the fragments are brought into apposition. A piece of silver wire is then inserted, the pieces held together, and the ends of the wire twisted several times, cut short and hammered down so that they lie flat along the bone. The arm may then be put up at right angles, since this is the most comfortable position for the patient; and an internal angular splint is as good as any because it does not press on the wound. Passive movement should be begun quite early, about the fourth or fifth day. Union is generally fairly firm after three weeks. If for any reason operation is considered inadvisable, the best splint to use is an anterior tin-splint bent at an angle of 160 degrees, and shaped to follow the curve of the arm. This is, of course, well padded and then fixed to the front of the arm by bandages which extend from the hand nearly to the shoulder. Passive movement cannot be begun so early as it can after wiring for fear of displacing the fragment. It is wise to allow a fortnight to elapse before beginning it. The reason for having a splint at that angle is that if the arm is bent more than that amount the triceps becomes taut, and pulls the olecranon backwards, whereas if the arm is extended beyond that angle, there is a danger of the radius and ulna becoming dislocated forwards. The disadvantages of the treatment are that the splint is a most inconvenient and irksome one to the patient, since the arm cannot be put into any form of sling to rest it, so that it is difficult to get about, and the greater part of the period of convalescence is necessarily spent on a couch. It is wise, if the patient will stand it, to keep the arm in this splint for five or six weeks. When passive movement is begun the arm is taken out of the splint at least every other day and rebandaged in it after the manipulations. But another and more serious disadvantage often follows splint treatment, as has been explained above, and that is fibrous union. When this has occurred the power of the triceps is diminished, because it is acting all the time at a disadvantage, since to all intents and purposes the muscle has been lengthened, and therefore it has to contract more than the normal amount to produce the normal result. This loss of power is sometimes so marked that complete extension of the arm is impossible, especially if the attempt be made against resistance. In a working man such disability will seriously impair his wage-earning capacity. To some extent the place of the weakened triceps can be supplied by an instrument which consists of a jointed armlet provided with a spring.

